# A randomized controlled trial assessing the safety and efficacy of palmitoylethanolamide for treating diabetic-related peripheral neuropathic pain

**DOI:** 10.1007/s10787-022-01033-8

**Published:** 2022-09-04

**Authors:** Emily Pickering, Elizabeth L. Steels, Kathryn J. Steadman, Amanda Rao, Luis Vitetta

**Affiliations:** 1 School of Pharmacy, University of Queensland, PACE Precinct, 20 Cornwall Street, Wooloongabba, Brisbane, QLD 4102 Australia; 2 Evidence Sciences Pty. Ltd., Brisbane, QLD Australia; 3 School of Human Movement and Nutrition Sciences, University of Queensland, Brisbane, QLD 4102 Australia; 4 Faculty of Medicine and Health, The University of Sydney, Sydney, NSW Australia

**Keywords:** Diabetic neuropathy, Pain, Neuropathic pain, Diabetes, Palmitoylethanolamide, PEA, Inflammation

## Abstract

**Background:**

Peripheral neuropathy is a common complication of diabetes. The management of the associated neuropathic pain remains difficult to treat.

**Objective:**

This study explored the safety, tolerability and efficacy of a palmitoylethanolamide (PEA) formulation in treating diabetic-related peripheral neuropathic pain (PNP). Secondary outcomes included systemic inflammation, sleep and mood changes in patients diagnosed with type 1 and type 2 diabetes and PNP.

**Design:**

This study was a single-centre, quadruple-blinded, placebo-controlled trial with 70 participants receiving 600 mg of PEA or placebo daily, for 8 weeks, with a 94% rate of study participation completion. Primary outcomes were neuropathic pain and specific pain types (the BPI-DPN and NPSI). The secondary outcomes were sleep quality (MOS sleep scale), mood (DASS-21), glucose metabolism and inflammation.

**Results:**

There was a significant reduction (*P* ≤ 0.001) in BPI-DPN total pain and pain interference, NPSI total score and sub-scores, except for evoked pain (*P* = 0.09) in the PEA group compared with the placebo group. The MOS sleep problem index and sub-scores significantly improved (*P* ≤ 0.001). DASS-21 depression scores significantly reduced (*P* = 0.03), but not anxiety or stress scores. Interleukin-6 and elevated C-reactive protein levels significantly reduced in the PEA group (*P* = 0.05), with no differences in fibrinogen between groups (*P* = 0.78) at treatment completion. There were no changes in safety pathology parameters, and the treatment was well tolerated.

**Conclusions:**

The study demonstrated that the PEA formulation reduced diabetic peripheral neuropathic pain and inflammation along with improving mood and sleep. Further studies on the mechanistic effectiveness of PEA as an adjunct medicine and as a monotherapy pain analgesic are warranted.

**Clinical Trial Registration:**

Registry name: Australian New Zealand Clinical Trials Registry (ANZCTR), Registration number: ACTRN12620001302943, Registration link: https://anzctr.org.au/Trial/Registration/TrialReview.aspx?id=380826, Actual study start date: 20 November 2020.

## Introduction

Diabetic peripheral neuropathy (DPN) is a neurodegenerative disorder of the peripheral nervous system. It is the most common complication of diabetes, estimated to affect up to 30% of this population, and is the leading cause of nerve pain, disability due to foot ulceration and amputation, disturbances to gait and fall-related injuries (Juster-Switlyk and Smith 2016). DPN affects the motor, sensory and autonomic nerves; however, it particularly targets the sensory nerves, which are responsible for the transmission of information including touch, temperature and injury pain (Bodman and Varacallo 2021). The characteristics of the nerve pain range from bouts of electric shocks and stabbing, abnormal sensations of tingling/pins and needles, spontaneous burning and increased sensitivity to pressure and evoked pain by brushing, pressure or cold temperatures (Bouhassira et al. 2004). These symptoms develop and worsen in the distal nerve endings of the feet and in severe instances spread towards the central parts of the body (Calcutt 2020). Small nerve fibres make up 79–91% of peripheral nerve fibres (Said et al. 1992; Malik et al. 2005) and are more sensitive and prone to damage than large nerve fibres. This results in the skin of the toes and fingers to be the first areas affected by DPN (Zochodne 2014). In DPN, continuously high serum glucose levels damage small blood vessels, causing reduced supply of oxygen and nutrients to the nerves (Bodman and Varacallo 2021). This compromises the myelin sheath insulating layer of the nerve fibres, particularly that of small nerve fibres, progressing to loss of integrity (Malik 2014). The ongoing damage to small nerve fibres contributes to the development of foot ulcerations (Gibbons et al. 2010), reduced vasodilation due to pressure ulcers (Koïtka et al. 2004) and impaired heat and pain perception (Malik 2014). A recent murine model of diabetes (Yang et al. 2019) suggested that variation to blood glucose levels weakens the myelin sheath and nerve fibres and induces inflammation, particularly increased pro-inflammatory cytokines such as tumour necrosis factor alpha (TNF-α), interleukin-6 (IL-6) and nuclear factor kappa-light-chain-enhancer of activated B cells (NF-_K_B). Consequently, the progressive reduction in the integrity of the myelin sheath has been demonstrated to lead to increasing severity of DPN (Malik 2014).

Maintaining control of blood glucose levels is a primary treatment strategy for DPN along with medications for the management of neuropathic pain which includes antiepileptics or anticonvulsants, tricyclic antidepressants, serotonin–norepinephrine reuptake inhibitors and opioids (Australian Medicines Handbook 2019). Reports demonstrate that PEA has potential as an analgesic treatment for DPN (D'amico et al. 2020). PEA belongs to the family of *N-*acylethanolamines (NAEs) that are endogenous biologically active lipid mediators synthesized on demand by the phospholipid membrane of cells (Alhouayek and Muccioli 2014; Mattace Raso et al. 2014). NAEs may assist in regulating pain and inflammation with a neuroprotective effect (Alhouayek and Muccioli 2014). PEA is also an endogenous ligand of the endocannabinoid system (ENCB) with neuromodulator activity in the central nervous system (Clayton et al. 2021). PEA has been reported to have an entourage effect which can enhance the physiological effects of the endogenous endocannabinoids such as N-arachidonoylethanolamine (i.e. anandamide). The overall effect is that through N-arachidonoylethanolamine and the ENCB system, anti-inflammatory and proapoptotic activities can inhibit pro-inflammatory markers such as TNF-α and NF-_K_B (Sancho et al. 2003). PEA is also considered to comprise a parallel endocannabinoid signalling system without the adverse effects such as those with exogenous endocannabinoids (e.g. THC).

Reduction in cellular PEA levels occurs due to prolonged inflammation (Solorzano et al. 2009) and as a result from nerve injury due to neuropathy (Franklin et al. 2003). An early meta-analysis with a diverse variety of chronic neuropathic pain conditions (three studies were specific for DPN) demonstrated that PEA was progressively effective in reducing chronic neuropathic pain with the report concluding that it has potential as a therapeutic strategy to manage chronic neuropathic pain (Paladini et al. 2016).

PEA, though, has been reported to be poorly bioavailable with oral-gut administration, hindering pharmacological efficacy (Gabrielsson et al. 2016). An increased absorbable form has been developed (Briskey et al. 2020) that has been reported to provide enhanced bioavailability and as such efficacy and dosing. The aim of the current clinical study was to determine whether an enhanced bioavailable formulation of PEA was safe, tolerable and effective for managing DPN-related pain and, moreover, and secondarily, this formulation was effective in reducing inflammation and improving quality of life (QOL) associated with DPN, over an 8-week study period in patients diagnosed with diabetes.

## Methods

### Trial design

The clinical study was an interventional, single-centre, prospective, randomized, quadruple-blinded, placebo-controlled, parallel study investigating the safety, tolerability and efficacy of a PEA formulation on neuropathic pain. The secondary outcomes of improvement in inflammation markers and sleep quality and improved quality of life when administered as an adjunct analgesic to diabetic medications were also measured over 8 weeks. The study included men and women aged at least 18 years of age with a diagnosis of type 1 or type 2 diabetes and who were prescribed glucose-lowering medications including either or both metformin and insulin. The study was conducted in Brisbane, Australia, in accordance with the principles of The Declaration of Helsinki and Australian Good Clinical Practice Guidelines. The trial was registered with the Australian New Zealand Clinical Trials Registry (ANZCTR) no.: ACTRN12620001302943.

### Investigational products

The investigational product was a proprietary formulation of palmitoylethanolamide (Palmidrol) which has previous approval by the Therapeutic Goods Association as an active ingredient in the listed medicines. The Palmidrol was provided by Gencor Pacific Ltd. and was combined with the bioavailability enhancement technology system LipiSperse® as defined by Briskey et al. (2022), under the brand name Levagen+. The investigational product was formulated and manufactured by Pharmako Biotechnologies Pty Ltd. under Good Manufacturing Practice guidelines. The investigational product was provided as clear capsules (size 00) containing 350 mg of Levagen+ providing no less than 300 mg Palmidrol per capsule, which contains excipients polyglycerol polyricinoleate (E476), coconut oil fractionated, lime oil, olive oil, lecithin (sunflower and/or oat) (E322), silica (E551), vitamin E), which were administered at a twice-daily dose, equating to a total of dosing of 600 mg/day of PEA over the 8-week study period. This dose has been shown to be gut absorbed to an equivalent dose of 1.1025 g of micronized PEA (Briskey et al. 2020). The matching placebo capsule contained 350 mg of maltodextrin. The capsules were packaged in high-density polyethylene (HDPE) bottles with a HDPE lid. The packaging, labelling and dosage administration of the placebo were the same as the investigational product.

### Participant inclusion and exclusion criteria

Participants meeting all inclusion and exclusion criteria were invited to participate in the clinical study. Males or females aged at least 18 years of age and diagnosed by a treating physician with type 1 diabetes or type 2 diabetes and with diabetic-related peripheral neuropathy were identified. Participants confirmed experiencing neuropathic pain by scoring more than four on the Neuropathic Pain Diagnostic Questionnaire (DN4) or scoring more than 12 on the Self-reported Leeds Assessment of Neuropathic Symptoms and Signs (S-LANSS). All participants were administering prescribed anti-diabetic medications metformin and/or insulin. Some participants (31/66, 47%) were also co-administering prescribed anti-diabetic medications with analgesic medications for pain. Participants were asked to continue taking their prescribed medications for the duration of the study and were permitted to take up to the maximum daily dose (4 g/day) of paracetamol as pain rescue medication.

Potential participants were excluded if the peripheral neuropathy was due to hereditary sensory neuropathy, vitamin B_12_ or folate deficiency, paraneoplastic diseases, advanced liver disease, kidney disease, hypothyroidism, prolonged phenytoin, warfarin or immunosuppressive drug use. Participants were also excluded if they were administering herbal medicines for pain relief including, but not limited to, turmeric/curcumin (*Curcuma longa)*, boswellia (*Boswellia serrata)*, willow bark (*Salix alba)* or medicinal cannabis. Women who were pregnant, planning to become pregnant or breastfeeding were excluded. Alcohol or substance abuse health issues, allergy or sensitivity to any of the ingredients in the investigational product or any clinically relevant abnormal findings, in the opinion of the investigators/clinicians, would make them not suitable for inclusion in the study. All participants that met all criteria provided written informed consent.

### Randomization, blinding and compliance

Eligible participants were randomly allocated to one of two study treatment groups with a ratio of 1:1. The study treatments with PEA or placebo twice daily were provided to participants in identical bottles labelled 001-070 sequentially upon enrolment. Participants were independently randomized prior to treatment medications being provided to the clinic. The clinical trial was quadruple masked (i.e. participant, care provider, investigators, outcomes assessor) to the allocation group of medications. Participant compliance was assessed by the number of capsules taken, with greater than 80% of the capsules consumed being accepted as compliant with doses administered.

### Clinical study outcomes

The primary outcome investigated the safety, tolerability and effectiveness of PEA administered concurrently with prescribed diabetic and pain medications to alleviate diabetic-related neuropathic pain. The secondary outcomes included a reduction in inflammatory markers and improvement in mood and sleep quality.

The primary outcome was considered as the overall severity of neuropathic pain, assessed with the Brief Pain Inventory Short Form for Diabetic Peripheral Neuropathy (BPI-DPN). The BPI-DPN comprised a four-item Pain Severity Score that rated worst pain, least pain, average pain and present pain with a numerical rating score of 0 (i.e. no pain) to 10 (i.e. worst imaginable pain). Pain was assessed at baseline, 2, 4, 6 and 8 weeks. Additionally, the BPI-DPN also contains a 10-item Pain Interference Score, which was also measured at baseline, 4 and 8 weeks as a concomitant primary outcome (Zelman et al. 2005). Additional pain associated outcomes included the severity of the specific characteristics of pain. The severity of the specific characteristics of pain was assessed using the Neuropathic Pain Symptom Inventory (NPSI) at baseline, 4 weeks and 8 weeks. The NPSI includes 12 items that allow discrimination and quantification of five distinct clinically relevant dimensions of neuropathic pain syndromes, namely superficial spontaneous burning, spontaneous pressing pain, paroxysmal pain (stabbing, pins and needles), evoked pain (mechanical brushing), thermal allodynia (pressure)/hyperalgesia (contact with cold) and dysesthesia/paraesthesia (pins and needles, tingling). Each question provides a numerical rating score of 0 (i.e. no pain) to 10 (i.e. worst imaginable pain) (Bouhassira et al. 2004).

Impact on sleep patterns was measured at baseline, 4 and 8 weeks using the Medical Outcomes Study—Sleep Scale (MOS). The MOS has been validated in patient populations experiencing neuropathic pain (Hays et al. 2005; Rejas et al. 2007). The MOS sleep score includes a Sleep Problem Index and the following dimensions of sleep disturbance: experience disturbance to sleep, achieving adequate sleep, sleep quantity, daytime somnolence during the day, occurrence of snoring and experiencing shortness of breath or headache upon awakening. The 21-item Depression Anxiety Stress Score (DASS-21) was also assessed at baseline and 8 weeks. This was a measure of mood distress along the three axes of depression, anxiety and stress (Ng et al. 2007).

Blood markers were assessed at baseline and at 8 weeks, and these included HbA1c and fasting blood glucose (FBG), as determined by the International Expert Committee guidelines (The Expert Committee on the Diagnosis and Classification of Diabetes Mellitus, 2003, The International Expert Committee 2009), and inflammation markers c-reactive protein (CRP), IL-6 and fibrinogen with normal reference ranges of 0–6 ml/L, 1.5–4.0 g/L and ≤ 1.8 pg/mL, respectively. Blood safety markers were assessed at baseline and 8 weeks and included a full blood count, liver function tests, platelets, electrolytes and kidney function. Product tolerability was assessed regularly at each clinic interview and during compliance checks by interviewers specifically documenting changes to treatment medications, new symptom concerns, new stressors or any mild, moderate or serious adverse events that ensued. An adverse event form in the case file forms was used to document adverse events. Adverse events (AEs) were defined as any unfavourable changes in health, including abnormal laboratory findings that occur in any clinical trial participant, primarily during the clinical trial period or within a specified period, following completion of the clinical trial. The safety testing procedures followed the NHMRC Safety Monitoring and Reporting in Clinical Trials guidelines (National Health and Medical Research Council 2016).

### Sample size

G*Power was used to calculate the sample size, based on a statistical difference (*P* < 0.05) between two independent means (active treatment and placebo groups, ratio 1:1) for the primary outcome (BPI-DPN Pain Severity Index). With a two-tailed, alpha error probability of 0.05 and a size effect of 0.8, the required total sample size was 29 participants per arm. Allowing for 15% rate of attrition, the required sample size for recruitment was inflated to a total of 70 eligible participants (in 1:1 ratio for active treatment group and placebo group (i.e. 35 per treatment group)). A previous effect size analysis of PEA for treatment of pain using the differences between days 0 and 21 of PEA treatment for visual analogue scale (VAS) scores from a meta-analysis (Clayton et al. 2021), with the assumption that the VAS scores were normally distributed, produced an estimated effect size (i.e. Cohen's *d*) value of 1.35 (95% CI 1.14–1.56) for the daily administration of 700 mg of micronized PEA (Gabrielsson et al. 2016).

### Statistical analysis

An intent-to-treat (ITT) approach was used for data analysis of the primary outcome, where all patients who were randomized to receive treatment and completed baseline and at least one subsequent assessment (including laboratory clinical markers) were included in the analysis. The ITT single missing data points in those completing the study were managed using the simple imputation method, with the data from the last observation carried forward. Normality between groups was tested using the Kolmogorov–Smirnov and Shapiro–Wilk tests of normality. Demographics including age, gender, weight, height and BMI were assessed for statistical differences between treatment groups at baseline and week 8 by Chi-square and presented with mean/SD. The pain scores were analysed for time and treatment effect using repeated measures ANOVA. The QOL questionnaires BPI-DPN, NPSI and MOS were analysed using t-tests (for normally distributed data) and with Mann–Whitney U test (for nonparametric data) between treatment groups. The DASS-21 and the laboratory markers were assessed by t-tests. Change scores were also used for data with wide variations in values and/or significant differences at baseline. Effect of confounders, including pain medications, was assessed using linear regression analysis. The SPSS version 27 software was used for statistical analyses and for generating the graphical figures. Statistical significance was set at a *P* < 0.05 (two-tailed).

## Results

### Participant demographics

A total of 235 prospective participants were recruited through public social media activities. Prospective participants registered their interest initially through an online screening form, followed by an e-consult of a comprehensive assessment including medical history and medications to establish eligibility. A total of 70 participants aged between 32 and 75 years met the inclusion and exclusion criteria and, following the provision of written informed consent, were enrolled in the study.

**Fig. 1:**
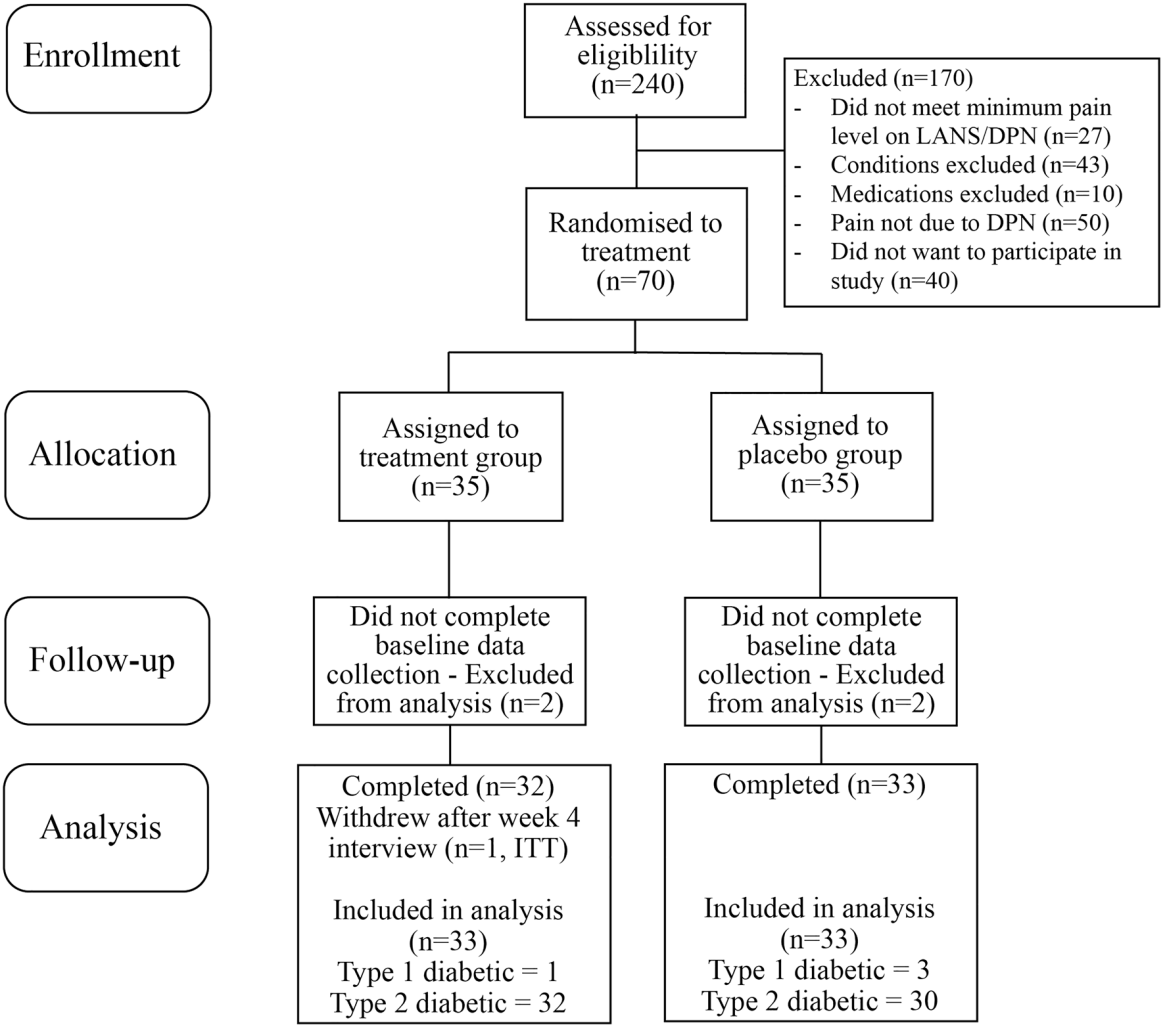
Participant progress CONSORT flow chart

The final analysis group included 66 participants (Fig. 1, Table 1), 33 in each arm. Four participants were excluded from analysis: three due to incomplete baseline assessments and one due to pre-existing (unknown) disease that was an exclusion criterion (Fig. 1). The cohort included three participants with type 1 and 62 with type 2 diabetes (Table 1). The groups had a different ratio of men to women, but there was no significant difference in baseline health parameters of body weight, BMI, blood pressure and lifestyle factors (Table 1). All participants were taking prescribed diabetic medications, and the majority were also taking prescribed pain medications as well as other medications for co-morbidities (Table 1).

**Table 1:** Baseline Characteristics

	Active group *n* = 35	Placebo group *n* = 35	*P* value^1^
Total (*n*) %	33 (50%)	33 (50%)	1.000
Men (*n*) %	20 (60%)	15 (40%)	0.22^1^
Women (*n*) %	13 (42%)	18 (58%)	
Total Age (Av, range)	65.5 [53–79]	61.5 [32–75]	0.056
Men Age (Av, range)	67.8 [53–79]	61.2 [32–75]	0.046
Women Age (Av, range)	62.1 [53–74]	61.8 [50–71]	0.92
Total BMI (Av, range)	31.2 [23.5–42.3]	31.5 [22.9–39.5]	0.80
Men BMI (Av, range)	29.9 [23.5–37.4]	31.4 [25.1–37.8]	0.27
Women BMI (Av, range)	33.3 [24.4–42.3]	31.7 [22.9–39.5]	0.39
Medications taken at baseline	*n* = 33	*n* = 33	
Metformin only	23	21	0.669
Insulin only	6	8	0.562
Metformin and Insulin	4	4	1.000
Additional diabetic medications^2^	19	22	0.626
Analgesic medications			
Ibuprofen	2	4	0.392
Paracetamol	7	7	1.000
Aspirin	1	3	0.302
Pregabalin	7	5	0.523
Other pain medications^3^	2	2	1.000
Tricyclic antidepressants^4^	4	4	1.000
Blood pressure medications	17	19	0.655
Cholesterol medication	16	14	0.621
Anti-depressant/anti-anxiety^5^	7	13	0.370

^1^Pearson Chi-Square test; all other *P* values assessed by two-tailed *t* test; statistical significance *P* < 0.05

^2^Additional diabetic medications: semaglutide, empagliflozin, linagliptin, dulaglutide, dapagliflozin, acarbose, gliclazide MR, vildagliptin, sitagliptin, exenatide

^3^Other pain medications: meloxicam (1) and tramadol (1), oxycodone (2)

^4^May have been prescribed for pain relief and/or mood support

^5^Includes tricyclic antidepressants (TCAs) which are also prescribed as a pain treatment for peripheral neuropathy

### Correlations between glucose metabolism, pain, sleep and mood

**Fig. 2:**
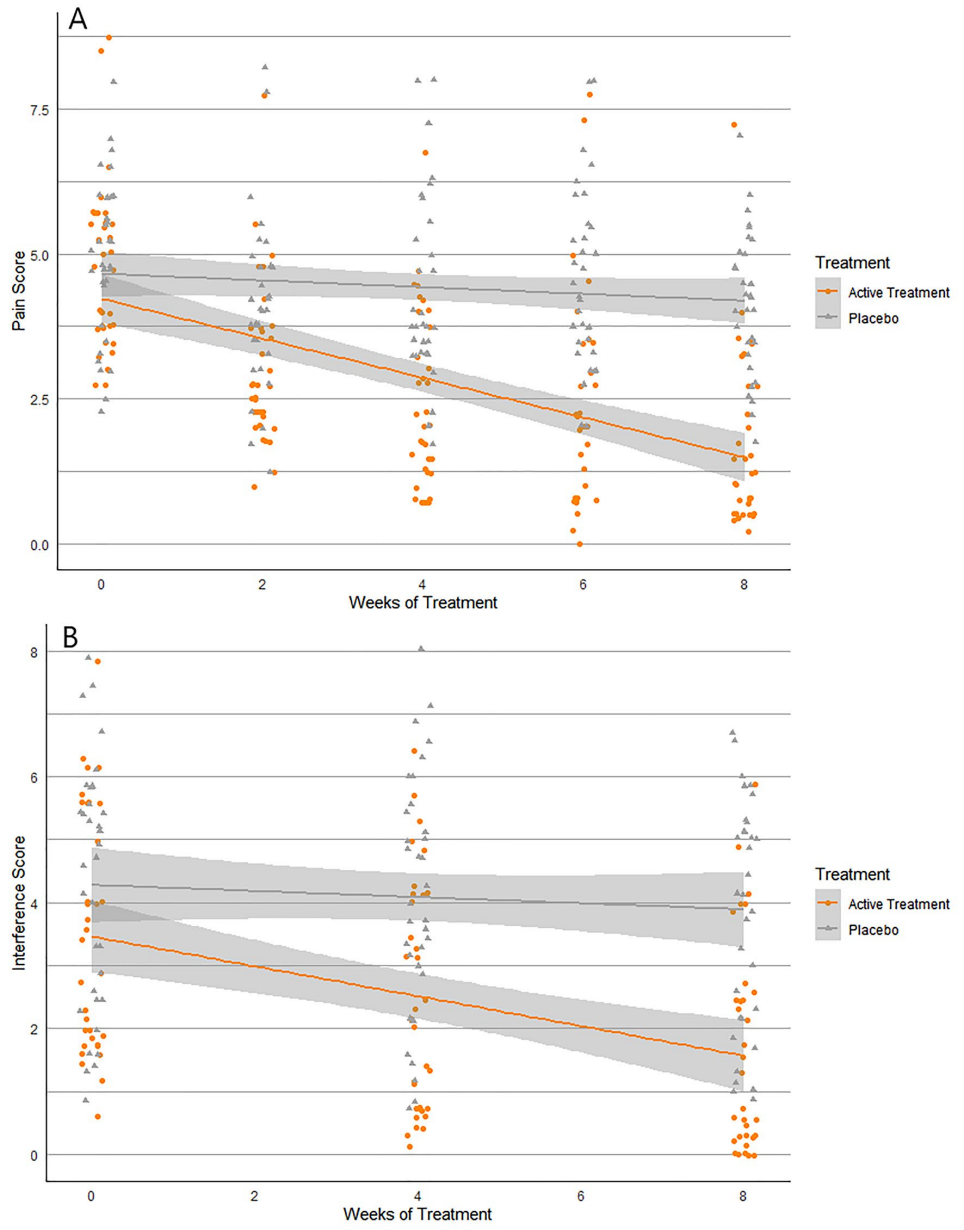
BPI-PN (A) Pain Severity scores at baseline, 2, 4, 6 and 8 weeks and (B) Pain interference score at baseline, 4 and 8 weeks scores for the active treatment group and the placebo group

**Fig. 3:**
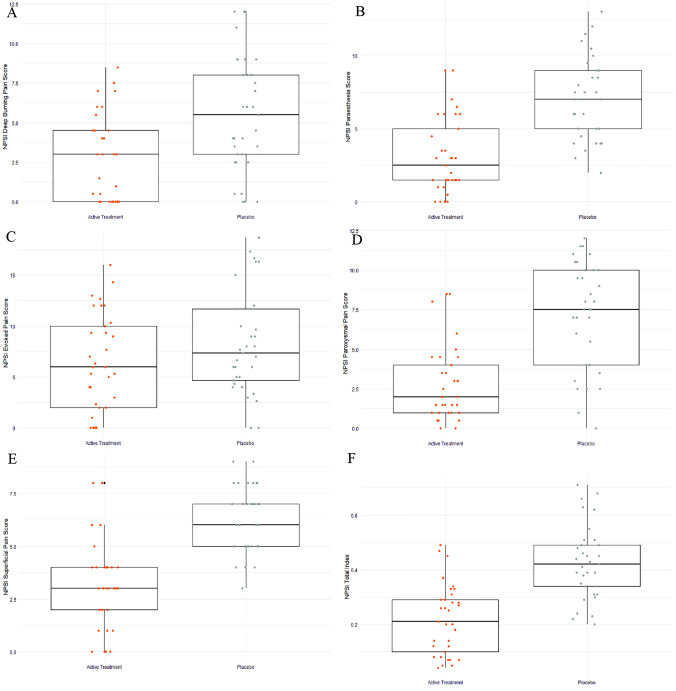
Neuropathic Pain Symptom Inventory (NPSI) total and sub-scores for the active treatment group and the placebo group at 8 weeks

There was a wide range in fasting blood glucose (FBG) and glycosylated haemoglobin (HbA1c) levels, despite the participants taking prescribed anti-diabetic medications (Table 3). The baseline FBG levels correlated with HbA1c levels (*r* = 0.662, *P* < 0.001). There were correlations between glucose metabolism and pain indices; particularly, a negative correlation between HbA1c and NPSI evoked pain (*r* = − 0.251, *P* = 0.045) and FBG and BPI-PN Pain Severity Index (*r* = − 0.322, *P* = 0.01), BPI-PN total pain (*r* = − 0.370, *P* = 0.003), the NPSI paroxysmal pain (*r* = − 0.266, *P* = 0.03), evoked pain (*r* = − 0.347, *P* = 0.005) and paraesthesia/dysesthesia (*r* = − 0.257, *P* = 0.04). Furthermore, CRP positively correlated with both NPSI deep pain (*r* = 0.261, *P* = 0.04) and fibrinogen levels (*r* = 0.387, *P* = 0.002). There was also a positive correlation between BPI-PN pain interference and depression scores (*r* = 0.431, *P* < 0.001), anxiety scores (*r* = 0.275, *P* = 0.03), stress scores (*r* = 0.270, *P* = 0.03) and sleep quality (*r* = 0.276, *P* = 0.03). Furthermore, CRP positively correlated with both NPSI deep pain (*r* = 0.261, *P* = 0.04) and fibrinogen levels (*r* = 0.387, *P* = 0.002) (Figs. 2 and 3).

### Effect of treatment on neuropathic pain severity and interference (BPI-DPN)

#### Pain severity

Pain severity as measured by the Brief Pain Inventory Short Form for Diabetic Peripheral Neuropathy (BPI-DPN) was not statistically different between groups at baseline, (*P* = 0.46) with most participants in both groups exhibiting mild to moderate pain (score of 2–7), (Table 2). Repeated measures ANOVA revealed a both time [*F* (4,64) = 21.03, *P* < 0.001] and treatment [*F* (1,64) = 23.52, *P* < 0.001] effect for the investigational product over 8 weeks of the study. Further analysis indicated that there was a significant difference in pain severity between groups at week 2 (*P* = 0.002), which continued at weeks 4, 6 and 8 (*P* < 0.001) (Table 2). Interestingly, a progressive and significant improvement in treatment group over placebo group in pain scores was observed as the study progressed.

**Table 2:** BPI-PN pain outcome scores for active treatment group and placebo group at baseline, 2, 4, 6 and 8 weeks

Intention to treat							
Outcome	Time Point	Group	Mean ± SD^1^	Range	*P* value	Change from baseline	Effect size [95% CI]
BPI Severity score^2^	Baseline	Active	4.77 ± 1.45	2.8–8.8	0.46		−0.135 [−0.617 to 0.349]
		Placebo	4.96 ± 1.37	2.3–8.0			
	Week 2	Active	3.03 ± 1.39	1.0–7.9	0.002	−1.75	−0.790 [−1.289 to −0.286]
		Placebo	4.14 ± 1.48	1.3–8.3		−0.80	
	Week 4	Active	2.49 ± 1.50	0.8–6.8	<0.001	−2.28	−1.131 [−1.648 to −0.607]
		Placebo	4.27 ± 1.64	1.8–8.0		−0.70	
	Week 6	Active	2.30 ± 1.84	0.0–7.8	<0.001	−2.47	−1.324 [−1.853 to −0.785]
		Placebo	4.54 ± 1.53	2.0–8.0		−0.42	
	Week 8	Active	1.72 ± 1.51	0.3–7.3	<0.001	−3.06	−1.811 [−2.381 to −1.231]
		Placebo	4.19 ± 1.20	1.8–7.0		−0.77	
BPI Interference score^2^	Baseline	Active	3.49 ± 1.90	0.6–7.9	0.15		−0.398 [−0.884 to 0.091]
		Placebo	4.26 ± 1.97	0.9–7.9			
	Week 4	Active	2.45 ± 1.85	0.1–6.4	0.001	−1.04	−0.882 [−1.385 to −0.373]
		Placebo	4.11 ± 1.90	0.7–8.0		−0.15	
	Week 8	Active	1.60 ± 1.66	0.0–5.9	<0.001	−1.90	−1.303 [−1.831 to −0.766]
		Placebo	3.87 ± 1.83	0.9–6.7		−0.39	

^1^Score given as average ± standard deviation

^2^Shapiro–Wilk distribution test found these data (in one or both arms) to be not normally distributed; tests of significance were performed nonparametrically with Mann–Whitney *U*. Active *n* = 33, Placebo *n* = 33. Statistical significance set at *P* ≤ 0.05

#### Pain interference

Pain interference as measured by the BPI-DPN was not significantly different between treatment groups at baseline (*P* = 0.15), (Table 2). When evaluating the effect of treatment on pain interference at week 4 and week 8, significant differences were observed between the two treatment groups [*F* (1,64) = 12.60, *P* ≤ 0.001] and the two time points [*F* (2,64) = 12.60, *P* ≤ 0.001]. At week 4, there was a significant difference between the means of the active treatment and placebo group (*P* = 0.001). Week 8 means also were significantly different between groups (*P* ≤ 0.001) with a greater change from baseline in the active group (−1.90) than the placebo group (−0.39).

### Effect of treatment on neuropathic pain symptoms (NPSI)

All measurements of neuropathic pain symptoms as measured by the NPSI were similar between groups at baseline (*P* ≥ 0.05, Table 3). By week 4, there was a significant difference between the active treatment and placebo groups for total pain score (*P* ≤ 0.001), superficial spontaneous pain (*P* ≤ 0.001), deep pain (*P* = 0.03), paroxysmal pain (*P* ≤ 0.001) and paraesthesia (*P* = 0.01). All these sub-scores were also found to be significantly different at week 8 for total pain (*P* ≤ 0.001), superficial pain (*P* ≤ 0.001), deep pain (*P* = 0.002), paroxysmal pain (*P* ≤ 0.001) and paraesthesia (*P* ≤ 0.001). The sub-score for evoked pain, however, was not significantly different between groups at week 4 (*P* = 0.25) yet was trending towards a significant difference by week 8 (*P* = 0.09).

**Table 3:** NPSI Pain outcome scores for active treatment group and placebo group at baseline, 4 and 8 weeks

Outcome	Group	Baseline	*P* value	4 weeks	*P* value	Change at 4 weeks	Effect size [95% CI]	8 weeks	*P* value	Change at 8 weeks	Effect size [95% CI]
Total pain score	Active	0.48 ± 0.17	0.86	0.29 ± 0.12	<0.001	−0.18	−1.061 [−1.574 to −0.541]	0.22 ± 0.13	<0.001	−0.25	−1.587 [−2.137 to −1.027]
	Placebo	0.47 ± 0.17		0.43 ± 0.13		−0.04		0.43 ± 0.13		−0.04	
Superficial pain	Active	5.94 ± 2.37	0.11	4.24 ± 2.15	<0.001	−1.70	−1.059 [−1.571 to −0.539]	3.21 ± 2.07	<0.001	−2.72	−1.651 [−2.207 to −1.086]
	Placebo	6.85 ± 2.18		6.21 ± 1.52		−0.63		6.24 ± 1.56		−0.60	
Deep pain	Active	5.79 ± 4.44	0.88^1^	3.68 ± 3.33	0.03	−2.10	−0.562 [−1.052 to −0.067]	2.79 ± 2.86	0.002^1^	−3.00	−0.841 [−1.342 to −0.334]
	Placebo	6.06 ± 4.45		5.79 ± 4.13		−0.27		5.55 ± 3.66		−0.51	
Paroxysmal pain	Active	8.65 ± 4.30	0.91^1^	4.29 ± 3.05	<0.001	−4.36	−0.991 [−1.500 to −0.476]	2.79 ± 2.35	<0.001	−5.86	−1.528 [−2.073 to −0.973]
	Placebo	8.56 ± 4.34		7.55 ± 3.51		−1.02		7.22 ± 3.37		−1.33	
Evoked pain	Active	8.99 ± 6.11	0.55^1^	7.02 ± 5.28	0.25	−1.97	−0.283 [−0.768 to 0.203]	6.27 ± 4.82	0.09	−2.72	−0.423 [−0.909 to 0.067]
	Placebo	8.25 ± 5.79		8.51 ± 5.19		0.25		8.39 ± 5.20		0.14	
Paraesthesia	Active	8.96 ± 3.02	0.20^1^	4.77 ± 3.00	0.01	−4.18	−0.642 [−1.135 to −0.145]	3.12 ± 2.57	<0.001	−5.83	−1.473 [−2.014 to −0.922]
	Placebo	7.91 ± 3.47		6.77 ± 3.22		−1.14		7.08 ± 2.79		−0.83	

Active *n* = 33, Placebo *n* = 33. Statistical significance set at *P* = < 0.05

^1^ Shapiro–Wilk distribution test found these data (in one or both arms) to be not normally distributed

Tests of significance are performed nonparametrically with Mann–Whitney *U* scores given as average ± standard deviation

### Influence of prescribed pain medications on treatment effect

The use of pain medication was not associated with any difference in BPI-PN pain severity, NPSI total and sub-scores at completion of treatment in either group. The mean pain medication index expressed as the number of non-treatment medication found no difference between treatment groups for total medications (prescribed and rescue) or rescue medications used.

### Effect of treatment on glucose metabolism and inflammatory markers

The treatment groups varied widely in glucose metabolism parameters (FBG and HbA1c) and the inflammatory markers (IL-6, fibrinogen and CRP) (Table 4). All markers were not significantly different at baseline (*P* > 0.05). At 8 weeks, there was no significant difference between the active treatment and placebo group for fasting blood glucose, HbA1c, fibrinogen and CRP when analysed as a whole group across all levels (*P* > 0.05). A sub-group analysis of participants with CRP levels of ≥ 5.0 mg/L at baseline was similar at baseline (*P* = 0.18); however, at 8 weeks the active treatment and placebo group demonstrated a significant difference between means (*P* = 0.05). For IL-6, the groups were also similar at baseline (*P* = 0.44); however, a significant difference was recorded at 8 weeks between the active treatment group and the placebo group (*P* = 0.04).

**Table 4:** Effect of treatment on glucose metabolism and inflammatory markers at for the active treatment group and placebo group at baseline and week 8

Test (Reference Range)	Group	Baseline Mean ± SD	Range	*P* value	8 Weeks Mean ± SD	Range	*P* value
Fasting Blood Glucose (3.0–7.7 mmol/L)	Active	7.91 ± 2.28	3.8–12.8	0.23	8.89 ± 3.17	5.1–20.2	0.79^1^
	Placebo	8.75 ± 3.26	3.3–19.0		9.13 ± 4.23	4.4–25.0	
HbA1c mmol/L (48–53 mmol/mol)	Active	58.35 ± 15.23	39.0–95.0	0.42	58.32 ± 16.47	37.0–96.0	0.53^1^
	Placebo	62.06 ± 20.73	36.0–121.0		62.97 ± 23.62	35–151	
HbA1c % (6.5–7%)	Active	7.49 ± 1.39	5.7–10.8	0.42	7.48 ± 1.51	5.5–11.0	0.53^1^
	Placebo	7.83 ± 1.90	5.4–13.3		7.91 ± 2.15	5.4–15.9	
C-reactive Protein (0–6 mg/L)	Active	5.32 ± 3.25	2.5–12.0	0.91^1^	4.63 ± 3.19	2.5–12.0	0.26^1^
	Placebo	5.97 ± 4.91	2.5–21.0		6.13 ± 4.74	2.5–18.0	
C-reactive protein > 5.0 mg/L	Active	8.38 ± 1.89	5.0–12.0	0.18	6.17 ± 3.35	2.5–12.0	0.05
	Placebo	10.13 ± 4.60	5.0–21.0		9.43 ± 5.07	2.5–18.0	
Interleukin-6 (< 6 pg/mL)	Active	4.93 ± 1.79	1.0–9.0	0.44	4.13 ± 2.32	1.0–12.0	0.04^**1**^
	Placebo	4.52 ± 2.27	1.0–12.0		5.44 ± 3.02	1.0–18.0	
Fibrinogen (1.50–4.00 g/L) ^1^	Active	3.73 ± 0.83	2.31–6.46	0.53^1^	3.55 ± 0.75	1.85–5.08	0.78
	Placebo	3.56 ± 0.85	2.15–4.93		3.61 ± 0.96	1.17–5.98	

Active treatment group *n* = 33, Placebo group *n* = 33. Statistical significance set at *P* ≤ 0.05

^1^Shapiro–Wilk distribution test found these data (in one or both arms) to be not normally distributed; tests of significance were performed nonparametrically with Mann–Whitney *U*

### Effect of treatment on sleep

The groups were similar at baseline for all the Medical Outcomes Study—Sleep Scale (MOS) subscale scores (*P* ≥ 0.05), (Table 5). At 8 weeks, there was a significant difference between the active treatment group compared with the placebo group in the sub-scores of sleep disturbance (*P* = 0.001), sleep adequacy (*P* = 0.001), daytime somnolence (*P* ≤ 0.001), shortness of breath or headache (*P* = 0.04) and the total sleep problem index (*P* ≤ 0.001). There was no significant change after 8 weeks for sleep quantity (*P* = 0.52) or snoring (*P* = 0.22).

**Table 5:** MOS sleep sub-scores for active treatment group and placebo group at baseline, 4 weeks and 8 weeks

MOS sub-scale	Group	Baseline Mean ± SD (range)	*P* value	4 Weeks Mean ± SD (range)	*P* value	Change at 4 weeks	Effect size [95% CI]	8 Weeks Mean ± SD (range)	*P* value	Change at 8 weeks	Effect size [95% CI]
Sleep disturbance*	Active	3.54 ± 0.79 (1.75–4.75)	0.64	3.92 ± 0.75 (2.00–5.00)	0.07^**1**^	0.37	0.458 [−0.033 to 0.945]	4.20 ± 0.56 (2.25–5.00)	**0.001**^**1**^	0.65	**0.843** **[**0.336 to 1.344]
	Placebo	3.64 ± 0.91 (1.75–5.00)		3.55 ± 0.84 (2.00–5.00)		−0.09		3.62 ± 0.79 (2.00–4.75)		−0.02	
Sleep adequacy*	Active	3.50 ± 1.47 (1.50–6.00)	0.39^1^	2.59 ± 1.27 (1.00–5.50)	0.04	−0.91	−0.507 [−0.995 to −0.014]	2.12 ± 0.91 (1.00–4.00)	**0.001**^**1**^	−1.38	**−0.831** **[−**1.331 to 0.324]
	Placebo	3.15 ± 1.29 (1.00–6.00)		3.24 ± 1.31 (1.00–5.50)		0.09		3.05 ± 1.28 (1.50–6.00)		−0.11	
Sleep quantity*	Active	6.02 ± 1.58 (2.00–1.00)	0.09	6.74 ± 1.43 (3.00–1.00)	0.69	0.72		6.83 ± 1.28 (4.00–9.00)	0.52	0.82	
	Placebo	6.67 ± 1.53 (4.00–1.00)		6.88 ± 1.38 (4.00–1.00)		0.21		7.04 ± 1.39 (4.00–1.00)		0.38	
Daytime somnolence*	Active	4.44 ± 1.14 (2.33–6.00)	0.11^1^	5.05 ± 0.89 (3.00–6.00)	0.001	0.61	0.923 [0.411 to 1.428]	5.30 ± 0.81 (3.33–6.00)	**<0.001**^**1**^	0.86	**1.178** [0.651 to 1.698]
	Placebo	3.90 ± 1.34 (1.00–6.00)		3.99 ± 1.36 (1.00–6.00)		0.09		4.08 ± 1.23 (1.00–6.00)		0.18	
Snoring**	Active	3.67 ± 1.90 (1.00–6.00)	0.07^1^	3.94 ± 1.85 (1.00–6.00)	0.43	0.27		3.91 ± 1.76 (1.00–6.00)	0.22^1^	0.24	
	Placebo	4.48 ± 1.66 (1.00–6.00)		4.39 ± 1.48 (1.00–6.00)		−0.09		4.45 ± 1.42 (2.00–6.00)		−0.03	
Shortness of breath or headache**	Active	5.76 ± 0.50 (4.00–6.00)	0.42^1^	5.82 ± 0.46 (4.00–6.00)	0.11	0.06		5.93 ± 0.24 (5.00–6.00)	**0.04**^**1**^	0.18	**0.540** [0.047 to 1.029
	Placebo	5.36 ± 1.32 (1.00–6.00)		5.36 ± 1.17 (2.00–6.00)		0.00		5.54 ± 1.00) (2.00–6.00)		0.18	
Sleep Problem Index**	Active	4.01 ± 0.35 (3.22–4.78)	0.25	4.12 ± 0.37 (3.11–5.00)	0.01	0.11		4.20 ± 0.29 (3.11–4.78)	**<0.001**	0.18	**0.859** [0.351 to 1.361]
	Placebo	3.82 ± 0.58 (2.11–4.67)		3.82 ± 0.53 (2.56–4.56)		0.0003		3.86 ± 0.46 (2.78–4.44)		0.04	

MOS score ratings depict the level of impact of each subcategory. Questions are a mix of positively (*) and negatively phrased (**) questions

^1^Shapiro–Wilk distribution test found these data (in one or both arms) to be not normally distributed; tests of significance are performed nonparametrically with Mann–Whitney *U*. Active treatment group *n* = 33, Placebo group *n* = 33. Statistical significance set at *P* ≤ 0.05

### Effect of treatment on the mood parameters depression, anxiety and stress

Groups were statistically different at baseline for depression (active: 2.06 ± 2.95, placebo: 3.97 ± 3.75, *P* = 0.02) and anxiety scores (active: 0.64 ± 1.34, placebo: 2.03 ± 2.89, *P* = 0.02), but not stress scores (active: 2.94 ± 3.46, placebo: 4.15 ± 3.41, *P* = 0.16), so analysis was performed on change scores. At 8 weeks, change scores were significantly different between the active treatment and placebo groups for depression (active: −1.27 ± 4.41 vs placebo: 0.09 ± 1.74, *P* = 0.02, *d* = −0.562, CI = −1.052 to −0.067) and were trending towards significance for stress (active: −1.36 ± 2.46 vs −0.39 ± 2.05, *P* = 0.09). There was no significant change in anxiety levels for either group by the end of 8 weeks (active: −0.333 ± 0.85 vs placebo: −0.545 ± 1.23, *P* = 0.42).

### Safety and tolerability of treatment

There were no changes observed for the independently reported laboratory markers for participant electrolytes, kidney and liver function. It is noteworthy, though, that blood glucose levels in both groups rose significantly over 8 weeks of the clinical trial period (change in fasting glucose from baseline: active + 0.88 mmol/L (+11.12%) *P* = 0.129, placebo + 0.95 mmol/L (+10.85%) *P* = 0.041, Table 4). Haematology parameters remained constant throughout the 8-week clinical trial except for a significant difference in the eosinophil count (0.04 vs −0.02, *P* = 0.01). There were several mild and short-duration adverse events reported by participants during the 8-week study period, which resolved over a few days without withdrawing from the study. These included intermittent mild headaches (*n* = 1, active treatment; *n* = 1, placebo), episodes of constipation (*n* = 1, active treatment), urticaria for 5 days (*n* = 1, active treatment), a severe fatigue episode (*n* = 1, placebo group) and respiratory infections not requiring additional medications (*n* = 3, placebo group) during the study. All adverse events resolved during the 8-week study period, and no participant was withdrawn from the study because of an adverse event.

## Discussion

The results of this study demonstrated that the formulation of PEA was safe, tolerable and demonstrated analgesic efficacy for mild to moderate neuropathic pain in patients diagnosed with diabetic-related peripheral neuropathy when tested over an 8-week period over placebo. The treatment reduced pain in different sensory nerves as shown by the reduction in spontaneous burning pain, spontaneous pressure pain, paroxysmal pain and paraesthesia with no relief observed from evoked pain, which is the response to touch, pressure and cold. Furthermore, the reductions in neurological pain were associated with lower levels of the inflammation markers IL-6 and CRP as well as a lower pain interference with life activities (i.e. walking, gardening, cooking, household duties), reduction in depression symptoms and improvement in quality of sleep.

The results of this study build on data from two previous clinical trials in patients diagnosed with DPN, albeit, with different doses of micronized PEA. It is notable that the analgesic effect of the PEA was significant from as early as 2 weeks. In a study that administered 600 mg of a micronized (non-emulsified) PEA, a significant reduction in neuropathic pain associated with DPN was observed after 30 days and 60 days (Schifilliti et al. 2014). Furthermore, the use of a higher dose of 1200 mg/day of the micronized (non-emulsified) PEA resulted in significantly reduced diabetic and traumatic chronic neuropathic pain after 40 days of treatment (Cocito et al. 2014). The results of the present study highlight the variability that can be encountered with different pain manifestations such as occurs with spontaneous, evoked, paroxysmal pain between individuals diagnosed with diabetes, which may reflect different mechanisms of the disease (Attal et al. 2008). In the current and previous studies, PEA was observed to have a significant effect in reducing the specific pain characteristic of paraesthesia/dysesthesia. PEA has also previously been shown to be effective in ameliorating pain associated with nerve compression syndromes such as sciatic pain (Keppel Hesselink and Kopsky 2015; Domínguez et al. 2012), carpal tunnel syndrome (Conigliaro et al. 2011) and Charcot–Marie–Tooth neuropathy (Putzu 2016) as well as knee osteoarthritis (Steels et al. 2019). The existing in-vitro, animal and human clinical studies demonstrate and support the efficacy of PEA in the treatment of algesia in neuropathies resulting from various causes (Paladini et al. 2016).

There is a strong correlation between pain and depression (Alghafri et al. 2020; Vas and Papanas 2020). The current study further confirms that PEA reduced depression symptoms when administered as an adjunct to citalopram (Ghazizadeh-Hashemi et al. 2018). Furthermore, we also report that the administration of PEA was associated with improvements in sleep adequacy, reduction in night-time sleep disturbances and daytime somnolence, confirming reports of improved overall sleep quality in surgical patients (Evangelista et al. 2018) and those patients with poor sleep quality, albeit without pain (Putzu 2016).

Prolonged hyperglycaemia observed in early or poorly controlled diabetes presents a metabolic disease with inflammatory sequelae and with upregulation of nuclear factor-KB (NF-_K_B), which subsequently increases TNF-α and drives a follow-on production of IL-6 and CRP (Yang et al. 2019; Mu et al. 2017, Patel and Santani 2009). Recent animal studies suggest that varying and elevated levels of blood glucose levels also weaken the myelin sheath and nerve fibres (Magrinelli et al. 2015). This process, alongside the production of inflammatory markers, is thought to progress the development of peripheral neuropathy (Jin and Park 2018). The inflammatory markers IL-6 and CRP were significantly reduced after treatment with PEA in this study, and it is suggested that reducing inflammation was part of the mechanism underlying the observed reduction in neuropathic pain. This is supported by recent cellular macrophage studies that have reported that PEA inhibits both TNF-α and IL-6 release (Del Re et al. 2021). It is noteworthy that there was a correlation in this study between elevated baseline fasting blood glucose and pain, also HbA1c and pain as well as CRP and pain. These data draw a link between effective management of blood glucose levels in pre-diabetics and diabetics with possible prevention and reduction in peripheral neuropathic pain.

A proportion of participants in this study were taking a range of prescribed anti-diabetic medications and medications to manage the neuropathic pain, which showed that PEA being effective as standalone treatment and an adjunct medication for pain relief. It was noteworthy that there was a significant placebo response observed, although PEA was significantly different to placebo, a phenomenon often seen in pain studies. Further research into effectiveness of PEA as an adjunct to long-term pain medication should be further investigated. There are limitations in this study that must be considered. The cohort was primarily type 2 diabetic and further research is needed with larger cohorts of both type 1 and type 2 diabetics. The cohort had varying levels diabetic control (as measured by HbA1c); therefor, larger studies may determine whether glycaemic levels impacted on the effectiveness of PEA. In addition, specific assessment of individual motor, sensory and autonomic nerve fibres is required to understand the full clinical potential of PEA in diabetic peripheral neuropathy.

## Conclusion

An 8-week randomized clinical study concluded that the PEA formulation investigated reduced diabetic-related neuropathic pain and was associated with a reduction in inflammation and depression. In addition, there was subsequent improvement in sleep quality. PEA was also well tolerated and reported to be safe as an adjunct in patients prescribed metformin and or insulin for the management of either type 1 or type 2 diabetes.

## Data Availability

Data described in the manuscript, code book and analytical code will be made available pending application and approval.

## Abbreviations

AEs: Adverse events
ANOVA: Analysis of variance
ANZCTR: Australian New Zealand Clinical Trials Registry
BMI: Body mass index
BPI-DPN: Brief pain inventory short form for diabetic peripheral neuropathy
CRP: C-reactive protein
DASS-21: 21-Item depression anxiety stress score
DPN: Diabetic peripheral neuropathy
DN4: Neuropathic pain diagnostic questionnaire
ENCB: Endocannabinoid
FBG: Fasting blood glucose
HbA1c: Glycosylated haemoglobin
HDPE: High-density polyethylene
IL-6: Interleukin-6
ITT: Intent to treat
MOS: Medical outcomes study—sleep scale
NAEs: *N-*Acylethanolamines
NF-_K_B: Nuclear factor kappa-light-chain-enhancer of activated B cells
NHMRC: National Health and Medical Research Council
NPSI: Neuropathic pain symptom inventory
PEA: Palmitoylethanolamide
PNP: Peripheral neuropathic pain
QOL: Quality of life
SD: Standard deviation
S-LANSS: Self-reported Leeds Assessment of Neuropathic Symptoms and Signs
SPSS: Statistical package for the social sciences
TNF-α: Tumour necrosis factor alpha
VAS: Visual analogue scale
